# Reusable vs single-use flexible cystoscopes in outpatient urology: a real-world micro-costing and user evaluation

**DOI:** 10.1186/s12894-026-02284-1

**Published:** 2026-07-28

**Authors:** Felix Seelemeyer, Rouvier Al-Monajjed, Jan Philipp Radtke, Stephan Nüesch, Jan Herden, Sassan Nazari, Ruben Gößmann

**Affiliations:** 1 Department of Urology, Medical Faculty, University of Düsseldorf, Düsseldorf, Germany; 2 Division of Personalized Early Detection of Prostate Cancer, German Cancer Research Center (DKFZ), Heidelberg, Germany; 3 Department of Radiology, German Cancer Research Center (DKFZ), Heidelberg, Germany; 4 Center for Management, School of Business and Economics, University of Muenster, Muenster, 48149 Germany; 5 International Institute of Management in Technology, University of Fribourg, Fribourg, Switzerland; 6 Department of Urology, PAN Clinic Cologne, Cologne, Germany; 7 Department of Urology, Medical Faculty, University of Cologne, Cologne, Germany

**Keywords:** Bladder cancer, Flexible cystoscopy, Single-use devices, Reusable devices, Micro-costing, Break-even analysis, Health economics

## Abstract

**Background and objective:**

Flexible cystoscopy is integral to outpatient urology, particularly for surveillance of non–muscle-invasive bladder cancer. Real-world economic data comparing reusable (RU) and single-use (SU) cystoscopes in German outpatient practice are scarce. We evaluated procedure-level costs and multidisciplinary user perceptions under routine conditions.

**Methods:**

A micro-costing analysis was performed in a German outpatient urology centre (337 cystoscopies/year). Capital investment (5-year amortisation), maintenance, reprocessing, consumables, and labour were included. Baseline and full-equipment replacement scenarios were modelled, and break-even thresholds calculated. A standardised survey of 13 physicians and 25 nursing staff from five practices assessed technical performance and workflow domains (5-point Likert scale). Groups were compared using Mann–Whitney U tests with Cliff’s delta.

**Results:**

Mean per-procedure cost was €27.25 for RU and €179.00 for SU. RU became cost-effective beyond 67 procedures annually; this threshold increased to 265 procedures when modelling full equipment renewal (€43,050). Physicians rated RU superior for manoeuvrability (*p* = 0.02) and SU superior for logistics (*p* = 0.02), with no differences in image quality, ergonomics, diagnostic confidence, or overall impression. Nursing staff favoured SU across all domains (all *p* < 0.001), particularly reprocessing workload and reliability. Limitations include single-centre design, evaluation of one device per category, context-specific reimbursement structures, and absence of primary environmental or patient-reported outcomes.

**Conclusions:**

In high-volume outpatient settings, RU cystoscopes are economically advantageous. However, SU devices offer substantial workflow benefits. Device selection should be context-specific, integrating case volume, infrastructure, and multidisciplinary perspectives.

**Supplementary Information:**

The online version contains supplementary material available at 10.1186/s12894-026-02284-1.

## Background

Flexible cystoscopy is one of the most frequently performed urological procedures and is central to the surveillance of non–muscle-invasive bladder cancer (NMIBC) In Germany, a substantial proportion of these procedures is undertaken in outpatient practices, where economic and organisational constraints are considerable. Under the statutory health insurance system, reimbursement according to the German Uniform Value Scale (Einheitlicher Bewertungsmaßstab, EBM) does not include device-related costs, in contrast to private insurance models that allow additional technical billing [1]. These structural differences create distinct financial implications for reusable (RU) and single-use (SU) cystoscopes.

Beyond reimbursement, reprocessing requirements and workflow efficiency influence device selection. RU cystoscopes require validated reprocessing, which is labour-intensive and associated with additional costs [2]. SU devices are supplied sterile and ready for immediate use, eliminating reprocessing steps but incurring higher per-procedure acquisition costs [3, 4]. Published economic evaluations of RU and SU cystoscopes have yielded heterogeneous results, often influenced by procedural volume and institutional structure [5].

To date, no context-specific real-world data from Germany directly compare RU and SU cystoscopes with regard to detailed micro-costing, workflow implications, and user perspectives under routine outpatient conditions. The present study therefore aimed to perform a practice-based cost–benefit analysis of RU and SU flexible cystoscopes in a German outpatient urology centre, integrating micro-costing data with structured assessments of physician and nursing staff perspectives.

## Methods

### Study design and setting

This real-world analysis was conducted in a German outpatient urology centre equipped with a dedicated cystoscopy suite. Flexible single-use (SU) and reusable (RU) cystoscopes were evaluated under routine diagnostic conditions, and procedures involving simultaneous double-J stent removal were excluded to ensure procedural comparability. The SU device examined was the Urofino™ SEEGEN UV-100-E (Shanghai SeeGen Photoelectric Technology Co., Ltd., Shanghai, China; distributed by Body Products Medizintechnik GmbH, Frechen, Germany) [6], while the RU comparator was the Olympus CYF-5 flexible cystoscope (Olympus Medical Systems, Tokyo, Japan) [7]. Both systems were used under identical organisational, technical, and hygienic conditions. Procedures involving simultaneous double-J stent removal were excluded to ensure procedural homogeneity, as these interventions require additional accessories and differ from routine diagnostic cystoscopy in procedural complexity, duration, and resource use.

### Economic evaluation and cost components

The economic evaluation followed a micro-costing approach including capital investment, maintenance and repair, reprocessing requirements, and labour expenditure Cost assumptions were based on original invoices, official distributor quotations, documented repair expenditure, recorded consumable use, measured reprocessing time, and gross personnel costs. As these inputs reflect the actual organisational structure of the study centre, they were considered context-specific rather than universally representative. To reduce the risk of underestimating reusable-system costs, an additional conservative scenario incorporating complete replacement of the reusable equipment was modelled. Moreover a deterministic one-way sensitivity analysis was performed using the annual break-even volume as the outcome. Reusable-system cost parameters were varied individually from 50% to 200% of their base-case values, while the single-use device price was varied by ± 25%; all other parameters were held constant. All costs were calculated net of tax. Capital costs were amortised linearly over a five-year period. Where original acquisition records for older reusable equipment were unavailable, acquisition costs were estimated using contemporaneous prices for technically equivalent models and verified against information provided by the manufacturer and authorised distributors. An additional scenario was modelled using an official distributor quotation for complete replacement of the RU inventory, including the camera head, processor with integrated light source, light cable, and HD monitor.

### Capital, maintenance, and reprocessing costs

Maintenance costs were derived from documented repair expenses during the observation period and extrapolated linearly. As SU systems do not require repair, no maintenance costs were assigned to them. A monitor supplied free of charge by the SU distributor was likewise excluded from calculations. Reprocessing costs for RU consisted of all consumables necessary for cleaning, disinfection, drying, leak testing, and documentation, including disinfectants, distilled water, brushes, and protective equipment. SU devices, being supplied sterile and ready for immediate use, incurred no reprocessing costs.

### Labour costs and per-procedure calculations

Labour costs were calculated based on the average documented reprocessing time of 20 min per procedure. Gross personnel costs were used to calculate labour expenditure. Total annual costs for all components of RU and SU usage were converted into per-procedure estimates using the centre’s annual case volume of 337 cystoscopies. To assess the effect of structural changes within a practice, scenarios modelling a complete switch to either RU or SU systems were additionally calculated.

### User survey design and participants

To complement the economic evaluation, a standardised user survey was conducted between August and October 2025 via the LimeSurvey platform. Participants included 13 urologists and 25 medical assistants from a regional network of five outpatient practices who regularly performed or assisted with flexible cystoscopy. Participation was anonymous and voluntary.

### Survey instruments and rating scales

The physician questionnaire assessed frequency of RU and SU use and subjective ratings of image quality, manoeuvrability and deflection, ergonomics, diagnostic confidence, logistics, and overall impression. The questionnaire for nursing staff focused on handling, reliability, logistics, reprocessing workload, and overall impression. All items were rated on a five-point Likert scale ranging from 1 (“very poor”) to 5 (“very good”), with optional free-text fields for additional comments. The questionnaires were study-specific instruments and were not externally validated. The full questionnaires are provided in Supplementary Appendix 1.

### Statistical analysis

Descriptive analysis included medians, interquartile ranges, and absolute frequencies. Group comparisons between RU and SU ratings were performed using the Mann–Whitney U test. Effect sizes were calculated according to Cliff’s delta with associated 95% confidence intervals. Multiple testing was controlled using the Benjamini–Hochberg procedure, although unadjusted p-values are reported for transparency. All statistical analyses were conducted using R version 4.4.1 (R Foundation for Statistical Computing, Vienna, Austria), and statistical significance was defined as *p* < 0.05.

## Results

### Cost analysis

Real-world expenditure data from August 2024 to August 2025 were used to calculate total and per-procedure costs for reusable (RU) and single-use (SU) flexible cystoscopes. A detailed breakdown of cost components for the RU system (Olympus CYF-5) is presented in Supplementary Table 1.

The RU system required an initial investment of €5515, which assuming a five-year amortisation period corresponded to annual capital costs of €1103 and €3.27 per procedure based on the annual case volume of 337 cystoscopies. Variable reprocessing costs averaged €12.86 per procedure, including €6.42 for disinfectants and €3.11 for distilled water. Maintenance and repair costs amounted to €5.45 per procedure. Labour for cleaning, disinfection, drying, and documentation totalled €5.67 per procedure, based on an average reprocessing time of 20 min.

Overall, mean cost per RU cystoscopy was €27.25.

**Fig. 1:**
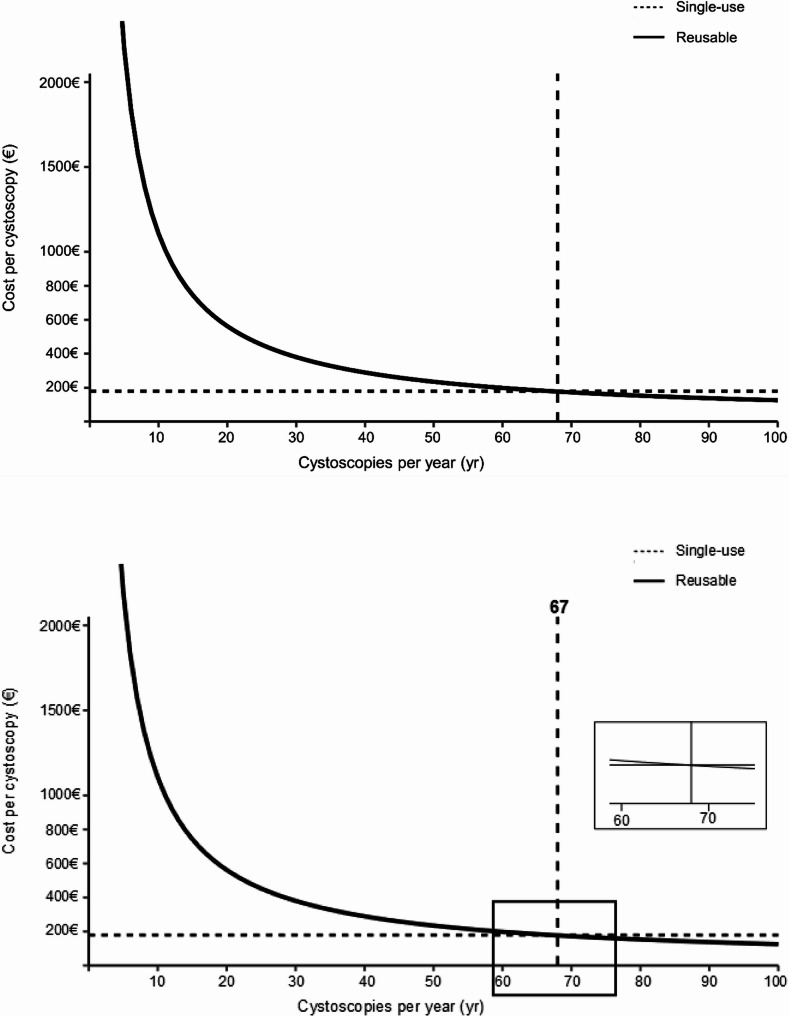
Break-even analysis of costs per flexible cystoscopy. The y-axis shows the estimated cost per cystoscopy (€), while the x-axis indicates the annual number of cystoscopies performed per year. The solid curved line represents the volume-dependent cost per procedure for reusable cystoscopes. The horizontal short-dashed line represents the cost of single-use cystoscopes, which remains constant because each procedure requires a new device at the same unit price, irrespective of annual case volume. The vertical long-dashed line marks the break-even point at 67 cystoscopies per year, above which reusable cystoscopes become more cost-effective. The boxed region surrounding the break-even point is shown at higher magnification in the inset

In contrast, SU cystoscopies (Urofino™ SEESGEN UV-100-E) generated uniform costs of €179.00 per procedure. The break-even analysis demonstrated that RU became more cost-effective once 67 procedures per year were exceeded (Fig. 1).

In the deterministic one-way sensitivity analysis, the base-case investment break-even threshold was 67.1 cystoscopies per year. The estimate was most sensitive to reusable-system acquisition costs, ranging from 50.2 procedures at 50% of the base-case value to 101.1 procedures at 200%. Varying the single-use device price by ± 25% yielded thresholds of 52.6–92.6 procedures. Changes in annual repair costs had a moderate impact, whereas reprocessing materials, gross hourly wages, and reprocessing time had limited effects. All modelled thresholds remained below the observed annual volume of 337 procedures (Supplementary Fig. 8.)

To account for variability in capital structures, an extended scenario incorporating a full replacement of all RU-related equipment (€43,050) was modelled. Under these assumptions, amortised capital costs increased to €25.55 per procedure, resulting in a total mean cost of €49.53 per RU cystoscopy. In this scenario, the break-even point shifted to 265 procedures per year (Supplementary Table 2, Supplementary Fig. 1). Given the study centre’s annual case load of 337 procedures, RU remained the more economical option in both models.

### Physician survey

Thirteen physicians completed the survey. Descriptive results are shown in Supplementary Tables 3, and inferential statistics in Table 1. Median ratings for both device types generally ranged from 3 to 5 on the five-point Likert scale. RU scores were consistently stable in logistics (median 3.0) and overall impression (median 4.0).

**Table 1:** Inferential results of the physician survey (*n* = 13)

Physicians Item	*n* (RU/SU)	*p*-value	Cliff´s delta (95%-CI)
Image quality	13/13	0.656	-0.10 (-0.52; 0.32)
Deflection	13/13	0.021	0.59 (0.22; 0.89)
Ergonomics	13/13	0.168	0.38 (-0.06; 0.76)
Diagnostic safety	13/13	0.355	0.26 (-0.15; 0.63)
Logistics	13/13	0.020	-0.63 (-0.89; -0.29)
Overall impression	13/13	0.493	-0.19 (-0.64; 0.28)

Shown are the results of Mann–Whitney U-tests with number of responses per group (n), *p*-values, and effect sizes according to Cliff’s delta with 95% confidence intervals

Inferential testing identified significant differences in two domains. Physicians rated RU significantly higher than SU in manoeuvrability/deflection (*p* = 0.021; Cliff’s delta = 0.59), indicating a moderate effect in favour of RU. Conversely, SU was rated significantly superior in logistics (*p* = 0.020; Cliff’s delta = − 0.63), reflecting a moderate effect in favour of SU.

No significant differences were observed for image quality, ergonomics, diagnostic confidence, or overall impression (all *p* > 0.05), and corresponding effect sizes were small with confidence intervals crossing zero.

Overall, physicians tended to favour RU for manoeuvrability, while valuing the logistical simplicity of SU.

### Nursing staff survey

Twenty-five medical assistants completed the nursing survey. Descriptive statistics are presented in Supplementary Tables 4, and inferential comparisons in Table 2. RU ratings ranged from 3 to 4 across domains, with broader variability compared to physicians. In contrast, SU consistently achieved median ratings of 5 and was predominantly scored in the upper range of the scale (4–7).

**Table 2:** Inferential results of the nursing staff survey (*n* = 25)

Nursing staff Item	*n* (RU/SU)	*p*-value	Cliff´s delta (95%-CI)
Handling	25/25	< 0.001	-0.71 (-0.88; -0.50)
Reliability	25/25	< 0.001	-0.88 (-0.98; -0.73)
Logistics	25/25	< 0.001	-0.90 (-0.99; -0.77)
Reprocessing effort	25/25	< 0.001	-0.95 (-1.00; -0.84)
Overall impression	25/25	< 0.001	-0.81 (-0.96; -0.62)

Shown are the results of Mann–Whitney U-tests with number of responses per group (n), p-values, and effect sizes according to Cliff’s delta with 95% confidence intervals

Inferential analysis demonstrated significant differences across all domains, each strongly favouring SU (all *p* < 0.001). Effect sizes according to Cliff’s delta ranged from − 0.71 to − 0.95, indicating large and consistent differences in favour of SU. The largest effect was observed for reprocessing effort (Cliff’s delta = − 0.95), suggesting a near-complete shift in rating distributions towards SU.

**Fig. 2:**
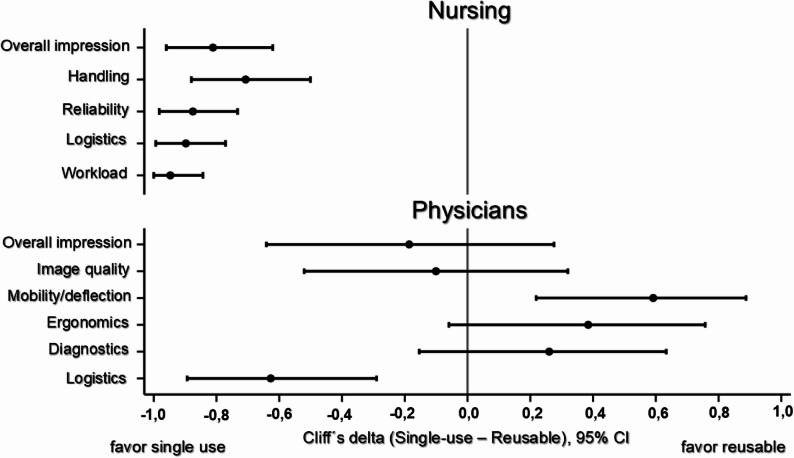
Forest plot of effect sizes comparing reusable and single-use cystoscopes. Forest plot displaying Cliff’s delta effect sizes with 95% confidence intervals for comparisons between reusable (RU) and single-use (SU) cystoscopes across physician and nursing staff ratings. Positive values favour RU and negative values favour SU. The vertical line at zero indicates no difference between device types

The magnitude and direction of effect sizes for both professional groups are summarised in the forest plot (Fig. 2), highlighting the divergence in domain-specific preferences.

### Supplementary analyses

Additional visualizations including heatmaps, radar plots and Likert scale ratings for both professional groups are provided in the Supplementary Figures (Supp. Figures 2–7). These graphics illustrate the relative weighting of user perceptions and further highlight the divergence between physicians and nursing staff.

## Discussion

### Economic considerations

In the present outpatient setting, reusable (RU) flexible cystoscopes were associated with substantially lower per-procedure costs than single-use (SU) devices (€27.25 vs. €179.00). The break-even threshold for RU was reached at 67 procedures per year, well below the annual case volume of the study centre (337 procedures), indicating clear cost superiority under current structural and financial conditions. Even when modelling full replacement of the RU equipment (€43,050), mean costs increased only to €49.53 per procedure, with a break-even point of 265 procedures per year, which remained below the observed case load. The sensitivity analysis confirmed that capital expenditure for the reusable system and the acquisition price of single-use devices were the main determinants of the break-even threshold. By contrast, even substantial variation in repair costs, reprocessing inputs, labour time, and gross wages had comparatively little effect.

Consistent with prior micro-costing analyses, the economic relationship between RU and SU cystoscopes is highly volume- and context-dependent. High-volume practices generally favour RU, whereas low-volume settings or environments with substantial fixed, service, or repair costs may shift the balance towards SU [5, 8, 9]. Model-based analyses further demonstrate that RU remain cost-competitive primarily under efficient, standardised reprocessing workflows, while urgent or non-standardised reprocessing can negate this advantage [10, 11].

Real-world data from a French single-centre study reported near cost equivalence between RU and SU (€196 vs. €192), underscoring the strong influence of local conditions on economic outcomes [12].

To facilitate comparison, an overview of published micro-costing studies comparing RU and SU flexible cystoscopes, including study design, reprocessing strategy, and per-procedure costs, is provided in Supplementary Table 5.

Reprocessing strategy represents a key determinant of cost. Studies incorporating centralised reprocessing and low-temperature sterilisation reported favourable economics for RU, whereas analyses relying on manual or AER-based high-level disinfection yielded less advantageous results [13, 14]. In contrast, prospective real-world data from US centres with predominant SU use reported lower per-procedure costs for SU, particularly when repair rates and backup requirements for RU were high [15].

Although the sensitivity analysis indicated that the economic advantage of RU persisted across the prespecified ranges, the findings remain context-specific and should be interpreted in light of the local cost structure, including refurbished equipment, the absence of service contracts, access to loan devices, and comparatively low reprocessing costs (€12.36 per procedure). Although direct reprocessing labour, consumables, and repair costs were included, the broader operational costs of RU cystoscopes may have been underestimated. Additional resource use related to disinfectant replacement, documentation, quality assurance, repair coordination, and device downtime was not separately quantified and may reduce the observed economic advantage of RU systems.

The SU monitor was provided at no additional charge under the current procurement agreement. However, such vendor-dependent arrangements are individually negotiated, may vary substantially between institutions, and may bundle hardware and service costs into the recurring device price. If practices were subsequently required to purchase or maintain the hardware independently, the long-term economic viability of the SU model could be reduced. These findings argue against generalisation of cost analyses and highlight the necessity of site-specific micro-costing. In addition, the German reimbursement framework materially affects economic evaluation. Within the german statutory health insurance system, cystoscopy is reimbursed as a fixed service without compensation for device-related costs, rendering the high unit price of SU particularly disadvantageous. By contrast, the private insurance system allows billing of technical equipment costs, potentially altering the economic viability of SU depending on patient mix. These structural differences create divergent incentives and should be considered in institutional decision-making [16]. In contrast to the payer-dependent German reimbursement framework, single-payer or bundled-payment systems, as reported in Canada and Singapore, assess device choice predominantly within fixed institutional budgets, making economic viability more directly dependent on total procedural costs, case volume, and local infrastructure [8, 11].

### User perspectives and workflow implications

Clinical performance remains a prerequisite in device selection. Current evidence indicates that modern SU cystoscopes are not inferior to RU devices in key clinical outcomes, including image quality, illumination, manoeuvrability, and procedural success [17]. Nonetheless, trade-offs exist: SU systems frequently demonstrate advantages in handling and reprocessing efficiency, whereas RU retain strengths in optical versatility and irrigation capacity [3]. In specific scenarios such as double-J stent removal, SU devices have shown superior performance due to integrated accessories [18]. In our study, procedures involving simultaneous double-J stent removal were excluded to improve procedural comparability, which may have obscured potential advantages of SU cystoscopes in accessory-assisted interventions. Large observational cohorts have reported no differences in oncological outcomes, including tumour detection and follow-up findings, supporting clinical equivalence in routine diagnostic use [19].

Safety considerations further inform this comparison. A meta-analysis of 21 studies identified no significant differences in complication or infection rates between SU and RU [20]. However, outbreaks of multidrug-resistant organisms linked to inadequate reprocessing of RU have been described [21], highlighting a potential infection-control advantage of SU. In addition, RU systems are subject to mechanical wear, with common failure points at distal tips and control mechanisms [22] whereas SU devices provide consistent performance without cumulative degradation.

Our survey findings reflect these multidimensional aspects and reveal a divergence between professional groups. Physicians rated RU superior in manoeuvrability but preferred SU for logistics, with no relevant differences in image quality, ergonomics, diagnostic confidence, or overall impression. However, these subjective user ratings do not establish clinical equivalence, as patient-level outcomes were not assessed. This suggests that RU may offer functional advantages in precision-demanding procedures, while SU are considered adequate for routine surveillance.

In contrast, nursing staff consistently favoured SU across all domains, with large effect sizes for reliability, handling, logistics, reprocessing workload, and overall impression. These results align with prior reports of improved workflow efficiency with SU, including shorter preparation and post-procedure times [23]. In outpatient practice, such efficiencies may translate into reduced staff burden, lower process complexity, and increased procedural capacity.

### Implications and contextualisation

In the present outpatient setting, subjective assessments of RU and SU cystoscopes were shaped not only by perceived technical performance but also by institutional context and professional perspective. Physicians tend to prioritize functional characteristics and procedural control, whereas nursing staff focus on workflow reliability, process safety, and reprocessing effort.

In high-volume outpatient settings with established RU infrastructure and predictable utilisation, RU systems remain the economically favourable choice. Conversely, SU cystoscopes may be advantageous in lower-volume or decentralised settings, during emergency or otherwise unplanned procedures requiring immediate access to a sterile device, where validated reprocessing capacity is limited, or when avoidance of reprocessing-related contamination is a particular concern. Device selection should therefore account not only for annual case volume and capital structure but also for immediate availability, reprocessing infrastructure, staffing, reimbursement conditions, infection-control priorities, and the functional requirements of the procedures performed. As patient-level outcomes such as pain, procedure duration, haematuria, urinary tract infection, antibiotic use, and patient satisfaction were not assessed, no conclusions regarding clinical equivalence can be drawn.

### Limitations

This study has several limitations. It was conducted in a single regional outpatient centre with a defined case volume, limiting generalizability to other healthcare contexts or international settings. Only one model per device category was evaluated: the Olympus CYF-5 and the Urofino™ SEEGEN UV-100-E. Given the potentially substantial variation in image quality, manoeuvrability, and other technical characteristics between single-use platforms, particularly across different manufacturers, the user-evaluation findings may not be transferable to other commercially available single-use cystoscopes.

Furthermore, cost structures such as reprocessing expenses and repair rates may differ substantially across institutions. Patient-level clinical outcomes, including procedural pain, procedure duration, postprocedural haematuria, urinary tract infection, antibiotic use, and patient satisfaction, were not assessed. Accordingly, the present findings cannot establish clinical equivalence between reusable and single-use cystoscopes and should be interpreted as an economic and user-perception comparison rather than a comparative effectiveness analysis.

The study-specific questionnaire was developed to capture device-related aspects of flexible cystoscopy, such as image quality, manoeuvrability, logistics, reliability, and reprocessing effort, which are not fully covered by generic validated instruments. However, because the questionnaire was not externally validated, the findings should be interpreted as exploratory assessments of user perception.

These limitations highlight the need for multicentre analyses and broader evaluations that account for structural variability.

## Conclusion

In this real-world analysis from a German outpatient urology centre, reusable cystoscopes (RU) demonstrated a clear economic advantage over single-use devices (SU), becoming cost-effective beyond 67 procedures annually and remaining favourable even under extended capital investment assumptions (break-even: 265 procedures). At the observed case volume, RU were unequivocally the more economical option.

User perspectives diverged between professional groups. Physicians preferred RU for manoeuvrability but rated SU superior in logistics, with no relevant differences in clinical performance domains Most notably, nursing staff strongly and consistently favoured SU cystoscopes across all evaluated domains, including handling, reliability, logistics, reprocessing workload, and overall impression, with large effect sizes. This pronounced preference highlights workflow efficiency and staff burden as clinically relevant determinants of device selection.

Device selection should therefore extend beyond cost considerations and integrate procedural volume, capital structure, reimbursement context, and multidisciplinary user priorities. While RU are economically advantageous in high-volume outpatient settings, SU may offer operational benefits in resource-constrained or lower-throughput environments.

Further multicentre studies in larger and more heterogeneous cohorts should incorporate life-cycle assessments to evaluate the environmental impact of RU and SU systems and support context-specific decision-making. Incorporating patient-reported outcomes and time-driven activity-based costing may further refine decision-making frameworks in outpatient urology.

## Supplementary Information

Supplementary Material 1.

## Data Availability

All data supporting the findings of this study are available within the paper and its Supplementary Information.

## Abbreviations

RU: Reusable
SU: Single–use
NMIBC: Non–muscle–invasive bladder cancer
CI: Confidence interval
AER: Automated endoscope reprocessor
HLD: High–level disinfection
CT: Computed tomography
DJ: Double–J (ureteral stent)
EBM: Einheitlicher Bewertungsmaßstab
