# Supplementary material for: Reusable vs single-use flexible cystoscopes in outpatient urology: a real-world micro-costing and user evaluation
Source: BMC Urol. 2026 Jul 28;26:180. doi: 10.1186/s12894-026-02284-1 (PMC13418758; doi:10.1186/s12894-026-02284-1)
Supplement: Supplementary file 1 — Supplementary Material 1. [file 12894_2026_2284_MOESM1_ESM.docx]

# Supplements

**
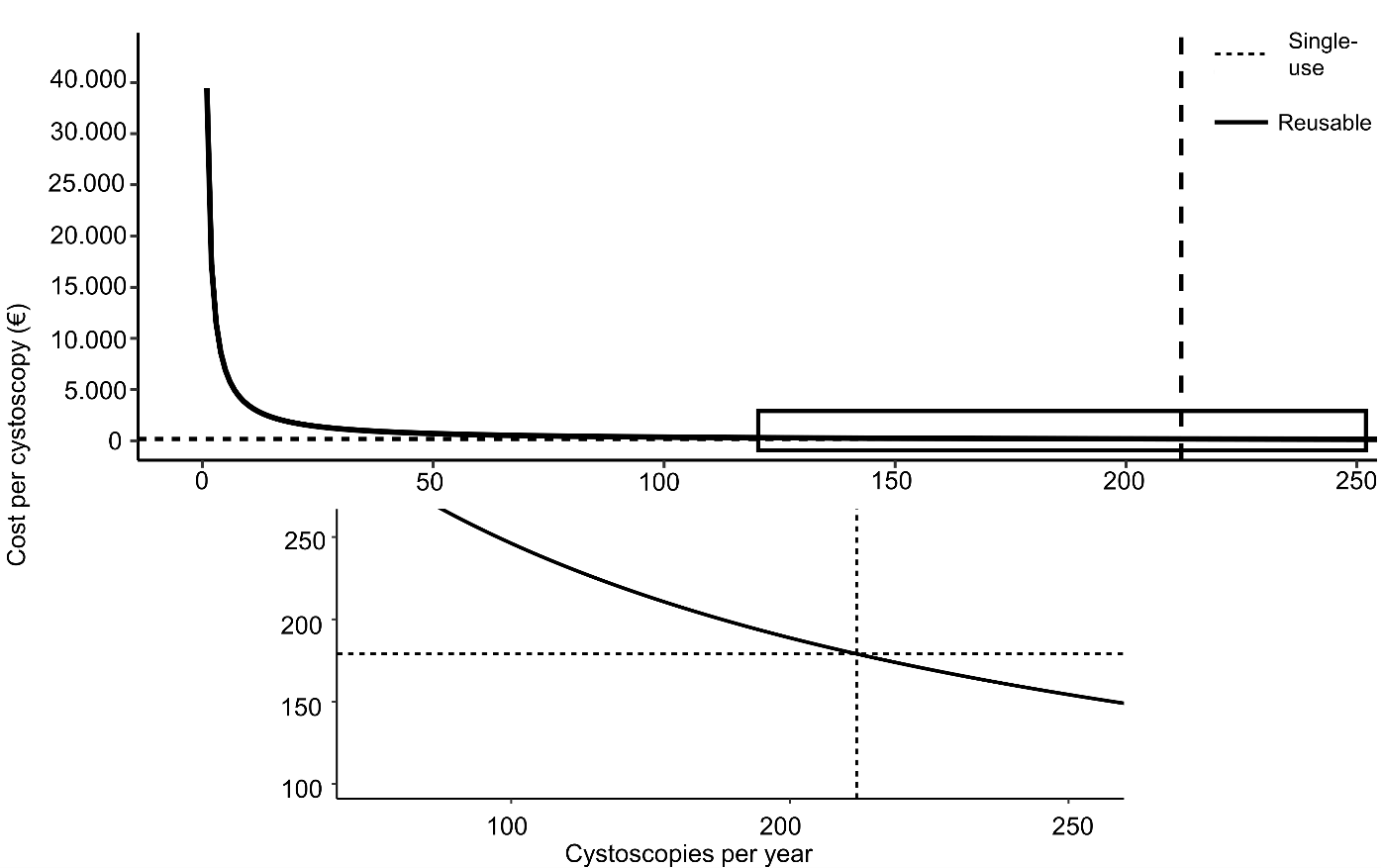
**

**Supplement Figure 1. Break-even analysis of costs per flexible cystoscopy including extended capital investment.**

The figure displays the calculated per-procedure costs for reusable cystoscopes (solid curved line) across varying annual case volumes, compared with the constant per-procedure costs of single-use devices (dashed line). Incorporating the extended capital investment scenario described in Table 2, the break-even point—at which reusable cystoscopes become more cost-effective than single-use alternatives—is reached at approximately 265 cystoscopies per year.

**
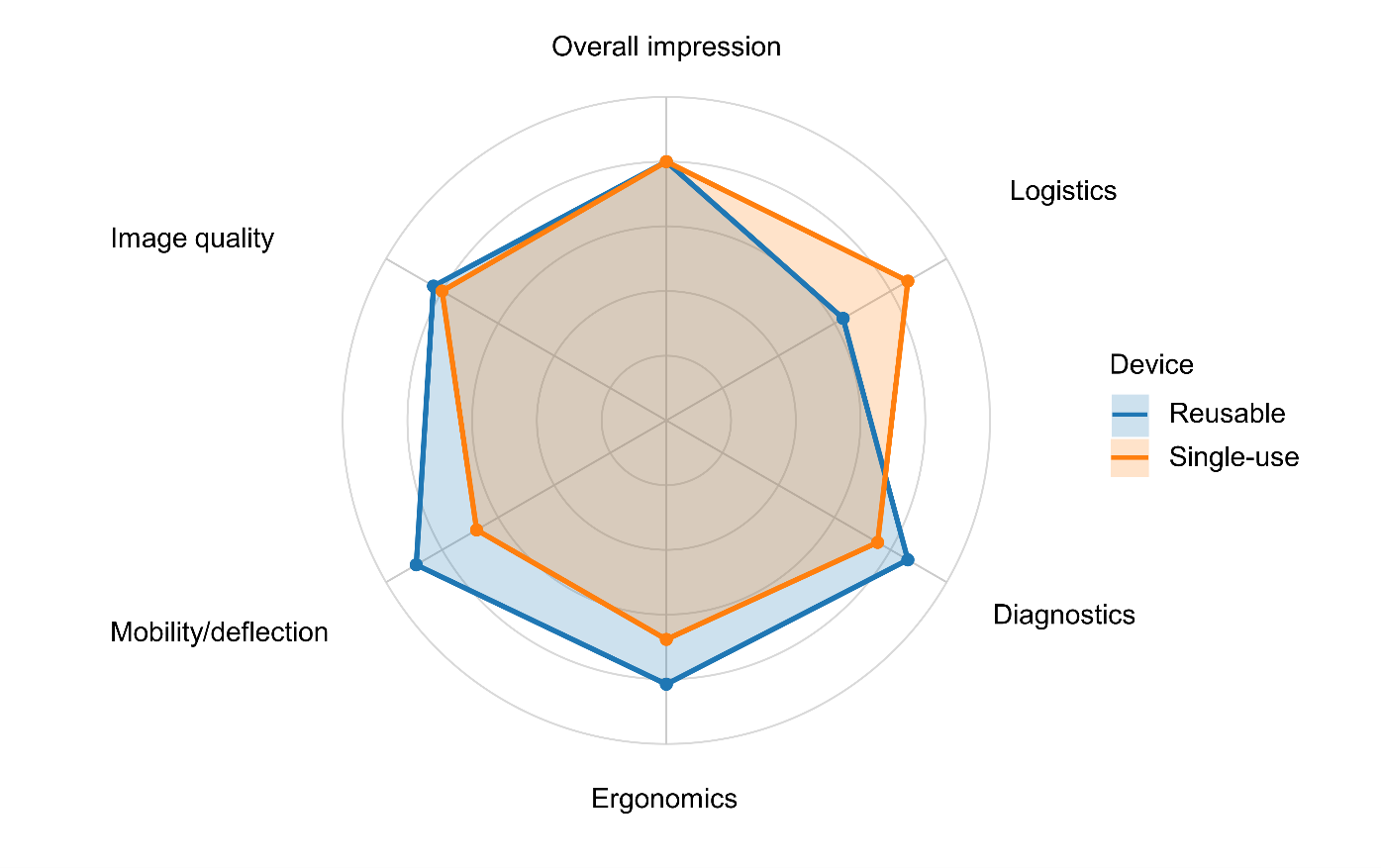
**

**Supplement Figure 2. Radar plot of mean physician ratings for reusable cystoscopes (blue) and single-use cystoscopes (orange).**

Displayed are the mean scores for six evaluation criteria: image quality, manoeuvrability, ergonomics, diagnostic confidence, logistics, and overall impression.

**
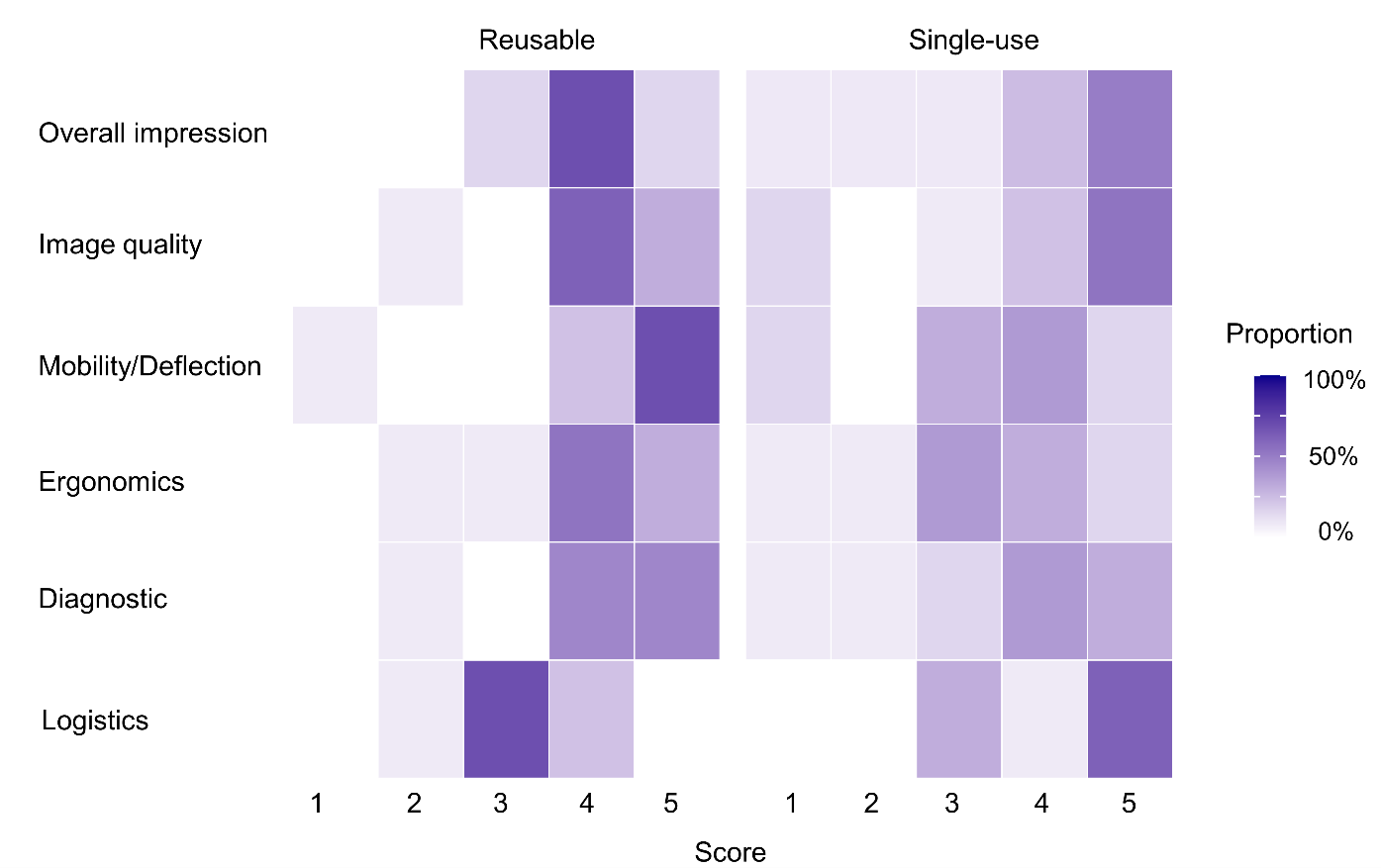
**

**Supplement Figure 3. Heatmap of physician ratings for reusable cystoscopes (left) and singleuse cystoscopes (right).**

Shown are the relative proportions of Likert scale ratings (1 = very poor to 5 = very good) for image quality, manoeuvrability, ergonomics, diagnostic confidence, logistics, and overall impression. Darker shades indicate higher proportions.

**
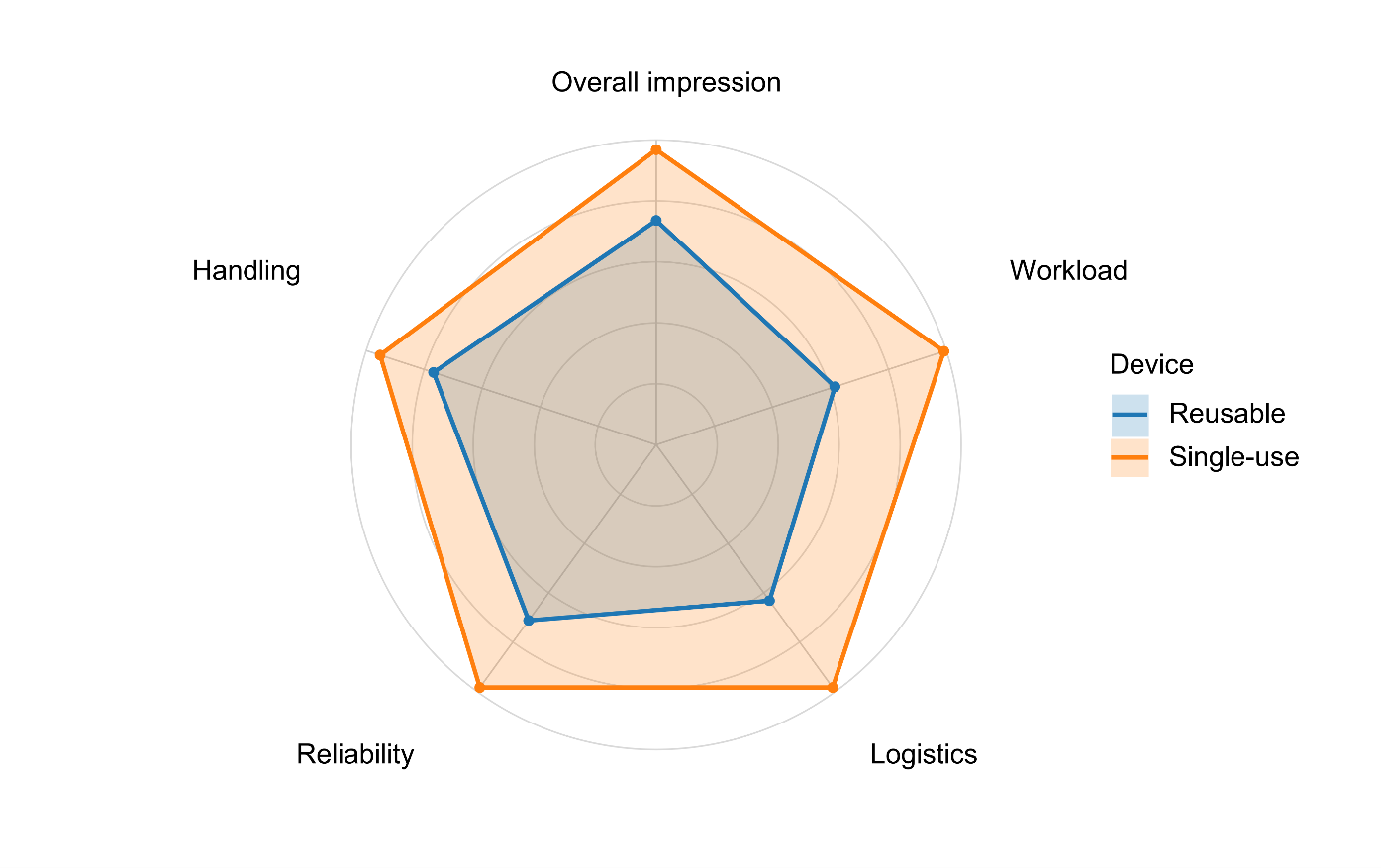
**

**Supplement Figure 4. Radar plot of mean nursing staff ratings for reusable cystoscopes (blue) and single-use cystoscopes (orange).**

Displayed are the evaluation criteria overall impression, handling, reliability, logistics, and reprocessing effort.

**
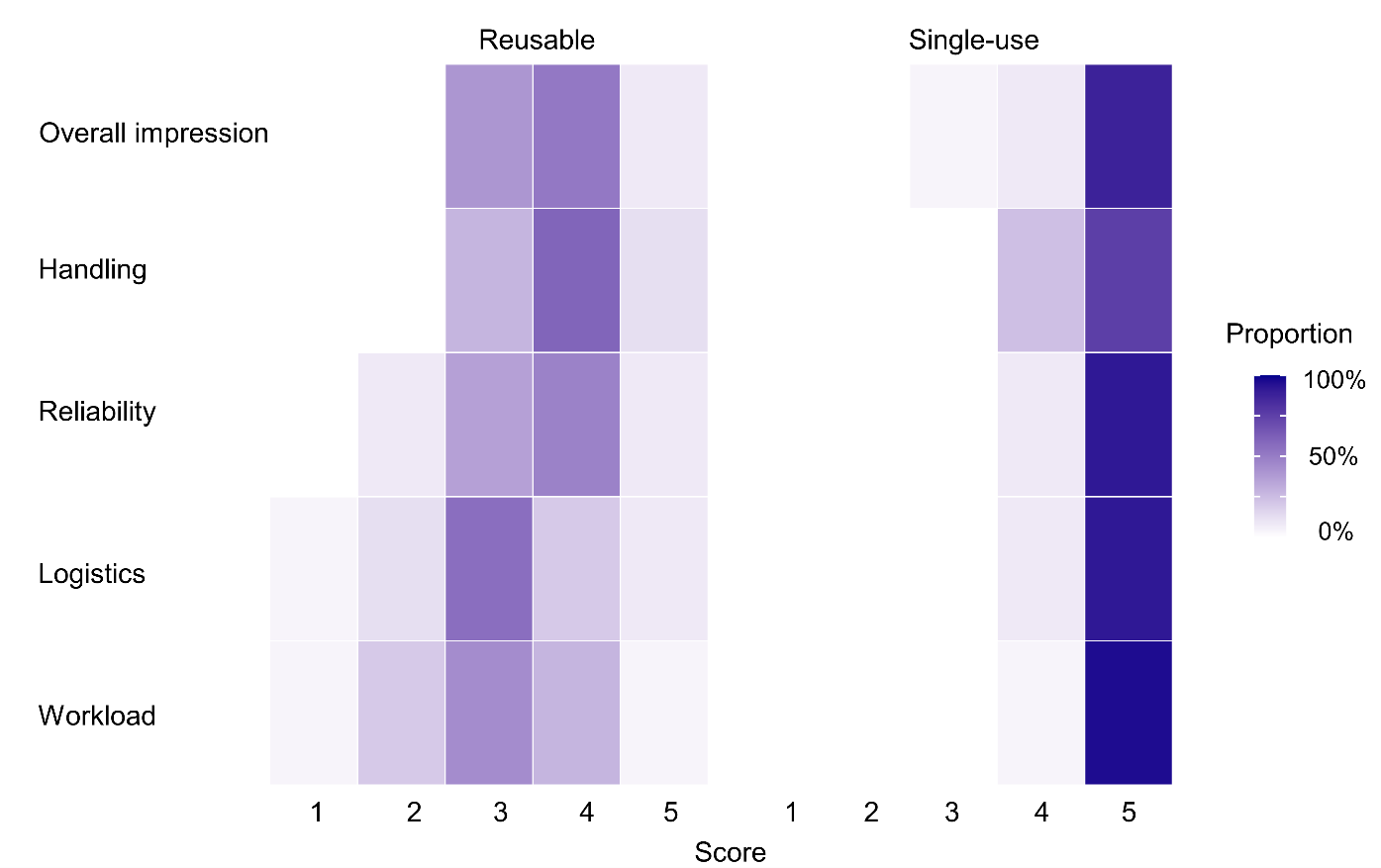
**

**Supplement Figure 5. Heatmap of nursing staff ratings for reusable cystoscopes (left) and single-use cystoscopes (right).**

Shown are the relative proportions of Likert scale ratings (1 = very poor to 5 = very good) for overall impression, handling, reliability, logistics, and reprocessing effort. Darker shades indicate higher response proportions.

**
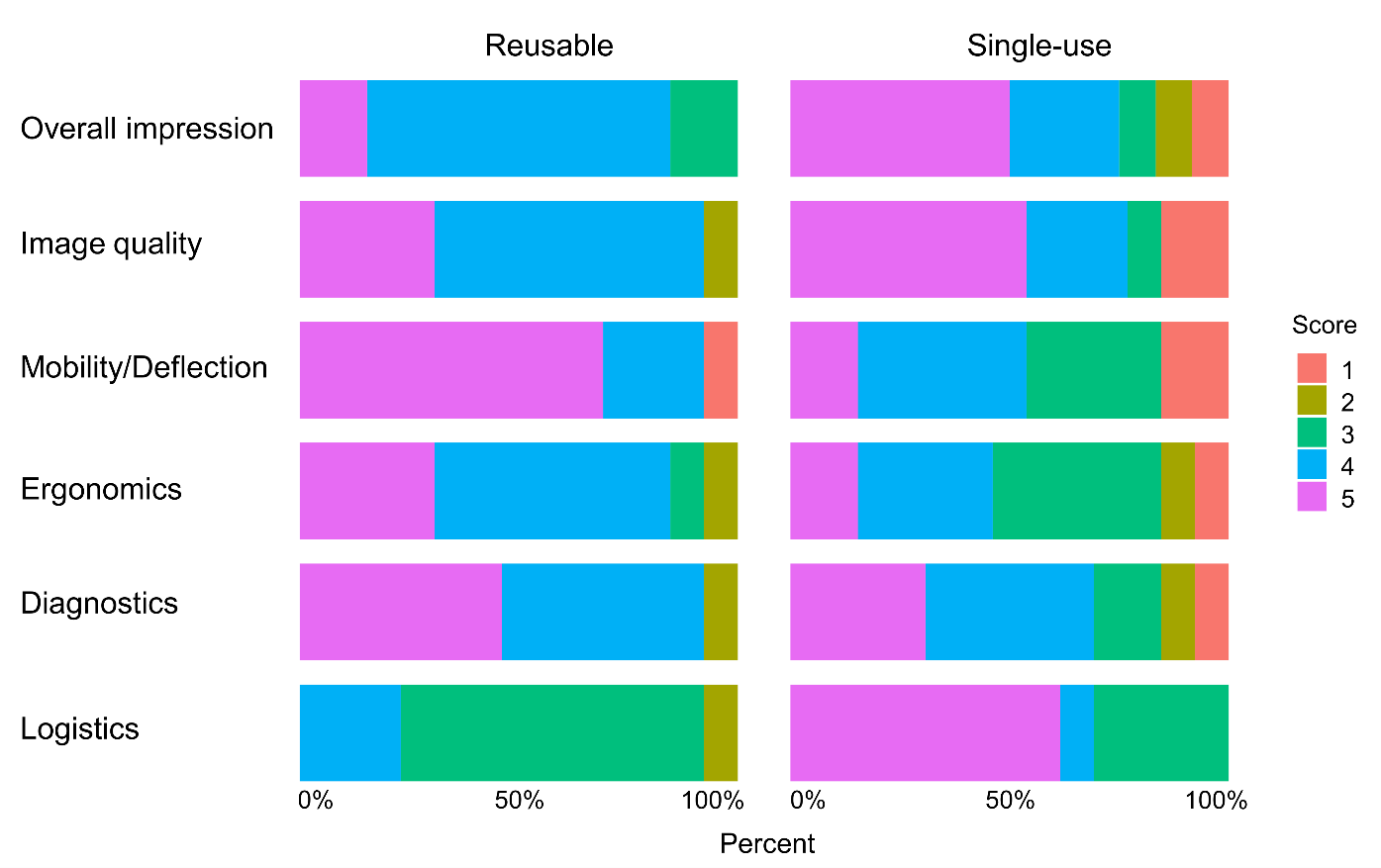
**

**Supplement Figure 6. Percentage distribution of physician ratings for reusable cystoscopes (left) and single-use cystoscopes (right).**

The figure illustrates the percentage distribution of Likert scale scores—1 = very poor (red), 2 = yellow, 3 = turquoise, 4 = blue, and 5 = violet—for image quality, manoeuvrability, ergonomics, diagnostic confidence, logistics, and overall impression. Each horizontal bar represents the relative proportion of ratings within the respective device group.

**
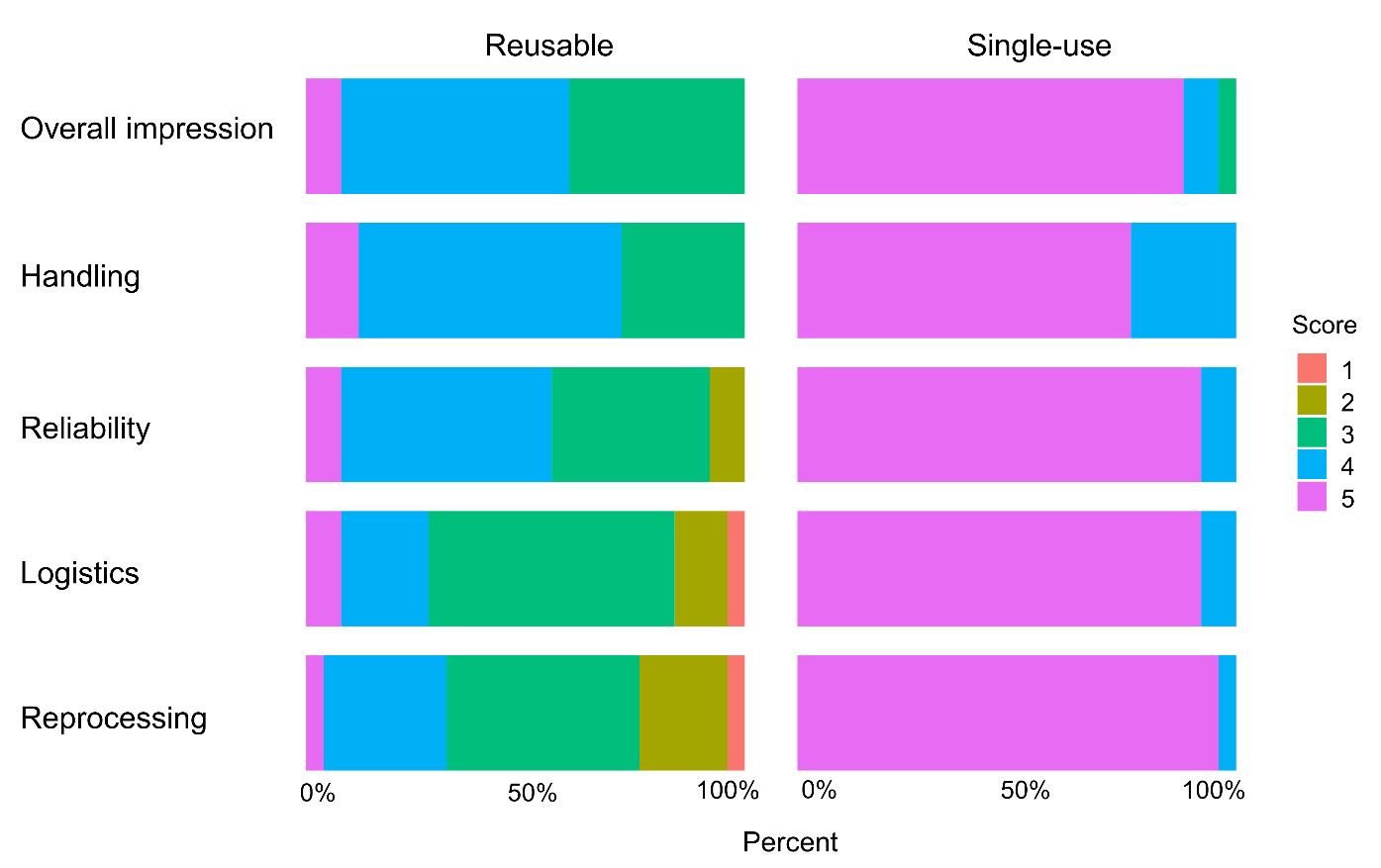
**

**Supplement Figure 7. Likert plot of nursing staff ratings for reusable cystoscopes (left) and single-use cystoscopes (right).**

The figure presents the percentage distribution of Likert scale scores—1 = very poor (red), 2 = yellow, 3 = turquoise, 4 = blue, and 5 = violet—for overall impression, handling, reliability, logistics, and reprocessing effort. Each horizontal bar depicts the relative proportion of ratings within the respective device group.


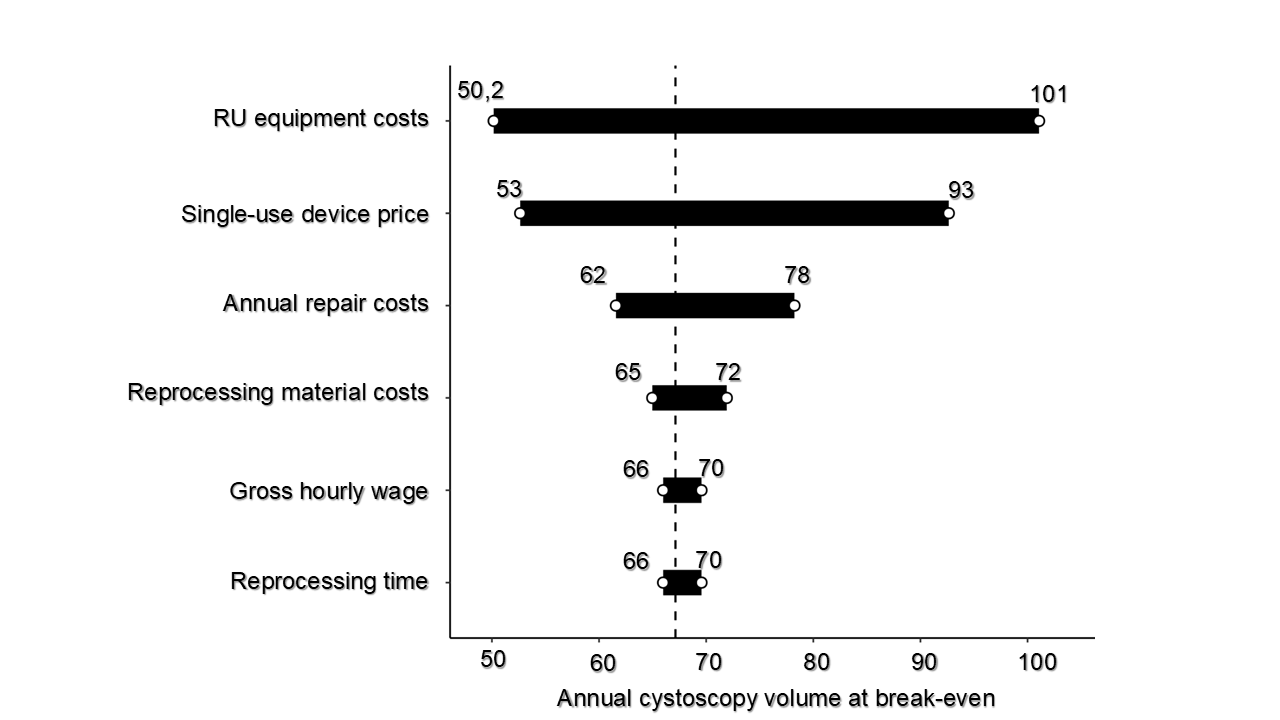


**Supplement Figure 8. Tornado plot of the deterministic one-way sensitivity analysis.** The x-axis indicates the annual cystoscopy volume at which reusable and single-use cystoscopy incur equal costs, and the y-axis lists the individually varied model parameters. The analysis used a break-even model separating fixed reusable-system costs from variable per-procedure costs and comparing these with the constant unit cost of single-use cystoscopy. Reusable equipment costs, annual repair costs, reprocessing material costs, reprocessing time, and gross hourly wages were varied from 50% to 200% of their base-case values, while the single-use device price was varied by ±25%. All remaining inputs were held at their base-case values. Horizontal bars represent the resulting range in break-even volume, and the dashed vertical line denotes the base-case threshold of approximately 67 procedures per year.

# Supplement Tables

| **Capital investment** Unit cost (€) | Amortization Annual cost (€) (years) | | | | Cost per cystoscopy (€) |
| --- | --- | --- | --- | --- | --- |
| Olympus CYF-5 cystoscope 3.665,00 € | 5 | | | 733,00 € | 2,18 € |
| Olympus AR-TF08E 500,00 € | 5 | | | 100,00 € | 0,30 € |
| Olympus OTV-SC digital signal processor 500,00 € | 5 | | | 100,00 € | 0,30 € |
| Karl Storz light cable | | 150,00 € | 5 | 30,00 € | 0,09 € |
| Karl Storz halogen light source 250 twin | | 600,00 € | 5 | 120,00 € | 0,36 € |
| LG monitor 15LC1R | | 100,00 € | 5 | 20,00 € | 0,06 € |
| Total capital costs | |  |  |  | *3,27 €* |
| **Maintenance**  Repair | | 1.804,64 € |  | 1.804,64 € | 5,36 € |
| Leak tester (Karl Storz) | | 150,00 € | 5 | 30,00 € | 0,09 € |
| **Reprocessing**  SICOLAB mini Endo, Dürr Technik | | 3.200,00 € | 5 | 640,00 € | 1,90 € |
| Sekusept aktiv, 6 kg powder | | 202,75 € |  | 2.162,00 € | 6,42 € |
| Reprocessing basins (3 units) | | 240,00 € | 5 | 48,00 € | 0,14 € |
| Distilled water, 21 L | | 5,25 € |  | 1.050,00 € | 3,12 € |
| Syringe, 10 ml (BRAUN) | | 0,06 € |  |  | 0,06 € |
| Cleaning brush | | 0,89 € |  |  | 0,89 € |
| Gauze compress, 10 × 10 cm | | 0,05 € |  |  | 0,05 € |
| Gloves, 100 units | | 0,06 € |  |  | 0,06 € |
| Cap | | 0,09 € |  |  | 0,09 € |
| Spray (Aesculap Sterilit Oil, 300 ml) | | 15,68 € |  | 31,36 | 0,14 € |
| Total reprocessing costs | |  |  |  | *12,86 €* |
| **Staff costs (20min)** | | 5,67 € |  |  | 5,67 € |
| **Total cost per cystoscopy** | |  |  | | **27,25 €** |

**Supplement Table 1. Overview of cost components per reusable flexible cystoscopy.**
Capital costs were amortised linearly over five years based on an annual case volume of 337 procedures. Reprocessing accounted for the largest share of total costs (€12.86 per procedure), followed by staff costs (€5.67), maintenance (€5.45), and capital investment (€3.27). The overall mean cost per reusable flexible cystoscopy was **€27.25.**

| **Capital investment** | Unit cost (€) | Amortization Annual cost (€) (years) | | Cost per cystoscopy (€) |
| --- | --- | --- | --- | --- |
| Olympus CYF-5 cystoscope | 7.600,00 € | 5 | 1.520,00 € | 4,51 € |
| HD pendulum camera head | 14.500,00 € | 5 | 2.900,00 € | 8,61 € |
| Video system incl. light source OTV-S500 | 17.500,00 € | 5 | 3.500,00 € | 10,39 € |
| Olympus light cable | 450,00 € | 5 | 90,00 € | 0,27 € |
| Olympus HD monitor 21" | 3.000,00 € | 5 | 600,00 € | 1,78 € |
| Total capital costs | *43.050,00 €* |  |  | *25,55 €* |
| **Maintenance**  Repair | 1.804,64 € |  | 1.804,64 € | 5,36 € |
| Leak tester (Karl Storz) | 150,00 € | 5 | 30,00 € | 0,09 € |
| **Reprocessing**  SICOLAB mini Endo, Dürr Technik | 3.200,00 € | 5 | 640,00 € | 1,90 € |
| Sekusept aktiv, 6 kg powder | 202,75 € |  | 2.162,00 € | 6,42 € |
| Reprocessing basins (3 units) | 240,00 € | 5 | 48,00 € | 0,14 € |
| Distilled water, 21 L | 5,25 € |  | 1.050,00 € | 3,12 € |
| Syringe, 10 ml (BRAUN) | 0,06 € |  |  | 0,06 € |
| Cleaning brush | 0,89 € |  |  | 0,89 € |
| Gauze compress, 10 × 10 cm | 0,05 € |  |  | 0,05 € |
| Gloves, 100 units | 0,06 € |  |  | 0,06 € |
| Cap | 0,09 € |  |  | 0,09 € |
| Spray (Aesculap Sterilit Oil, 300 ml) | 15,68 € |  | 31,36 | 0,14 € |
| Total reprocessing costs |  |  |  | *12,86 €* |
| **Staff costs (20min)** | 5,67 € |  |  | 5,67 € |
| **Total cost per cystoscopy** |  |  | | **49,53 €** |

**Supplement Table 2. Overview of cost components per reusable flexible cystoscopy under extended capital investment.**
Capital costs were amortised linearly over five years based on an annual case volume of 337 procedures. In this extended investment scenario, capital expenditure accounted for €25.55 per procedure, substantially higher than in the baseline model, while reprocessing (€12.86), staff (€5.67), and maintenance (€5.45) remained similar. The overall mean cost per reusable cystoscopy was **€49.53.**

| **Physicians**  **Item** | **Reusable** | **Single-Use** |
| --- | --- | --- |
| Image quality | 4.0 (4.0-5.0) [2-5]  0/1/0/8/4 | 5.0 (4.0-5.0) [1-5]  2/0/1/3/7 |
| Deflection | 5.0 (4.0-5.0) [1-5]  1/0/0/3/9 | 4.0 (3.0-4.0) [1-5]  2/0/4/5/2 |
| Ergonomics | 4.0 (4.0-5.0) [2-5]  0/1/0/6/6 | 3.0 (3.0-4.0) [1-5]  1/1/5/4/2 |
| Diagnostic safety | 4.0 (4.0-5.0) [2-5]  0/1/0/6/6 | 4.0 (3.0-5.0) [1-5]  1/1/2/5/4 |
| Logistics | 3.0 (3.0-3.0) [3-3]  0/0/13/0/0 | 5.0 (3.0-5.0) {3-5]  0/0/2/4/7 |
| Overall impression | 4.0 (4.0-4.0) [4-4]  0/0/0/13/0 | 4.5 (3.5-4.0) {3-5]  0/0/1/5/7 |

**Supplement Table 3. Descriptive results of the physician survey (n = 13 per arm) comparing reusable and single-use cystoscopes**

Data are presented as median (Q1–Q3), range [min–max], and absolute frequencies of Likert scale ratings (n1/n2/n3/n4/n5).

| **Nursing staff**  **Item** | **Reusable** | **Single-Use** |
| --- | --- | --- |
| Handling | 4.0 (3.0-4.0) [3-4]  0/0/9/16/0 | 5.0 (5.0-5.0) [4-5]  0/0/0/2/23 |
| Reliability | 4.0 (3.0-4.0) [3-4]  0/0/6/19/0 | 5.0 (5.0-5.0) [5-5]  0/0/0/0/25 |
| Logistics | 3.0 (3.0-4.0) [3-4]  0/0/13/12/0 | 5.0 (5.0-5.0) [5-5]  0/0/0/0/25 |
| Reprocessing effort | 3.0 (3.0-4.0) [1-5]  1/5/11/7/1 | 5.0 (5.0-5.0) [4-5]  0/0/0/1/24 |
| Overall impression | 4.0 (3.0-4.0) [3-5]  0/0/10/13/2 | 5.0 (5.0-5.0) [3-5]  0/0/1/2/22 |

**Supplement Table 4. Descriptive results of the nursing staff survey (n = 25 per arm) comparing reusable and single-use cystoscopes**

Data are presented as median (Q1–Q3), range min–max, and absolute frequencies of Likert scale ratings (n1/n2/n3/n4/n5).

| Author | Country | Study design | RU System | SU system | Reprocess-ing | Cost RU | Cost  SU | Advantage |
| --- | --- | --- | --- | --- | --- | --- | --- | --- |
| Franzoso 2025  (11) | Italy | Retrospective (model-based) | Reusable modelled | manufacturer not specified | Modelled | €181-199€ | €189 | Context-dependent |
| Foo 2025 (12) | Singapore | Retrospective | Olympus CYF-5 | manufacturer not specified | HLD (AER) | ≤ SGD 135 | typically > SGD 200 | Reusable |
| Kim 2022 (9) | Canada | Retrospective | Olympus CYF-VH | Ambu aScope | LTS | CAD 149 | CAD 246 | Reusable |
| Ait Taleb 2025 (14) | France | Retrospective | KARL STORZ  ref. 11272 | Ambu a 4 C | LTS | €136 | €192 | Reusable |
| Bertolo 2024 (15) | Italy | Retrospective | KARL STORZ, Olympus (model not specified) | Ambu aScope | HLD (AER) | €332 | €220 | Single-use |
| Kenigsberg 2021 (10) | USA | Retrospective | manufacturer not specified | theoretical cost model | HLD | USD 133-161 | USD 222 | Reusable |
| Assmus 2022 (16) | USA | Prospective | manufacturer not specified | Ambu aScope 4 C | HLD | USD 272 | USD 185 | Single-use |
| Young 2021 (6) | USA | Retrospective | Olympus CYF-VHR, CYF-V2 | Ambu aScope 4 C | HLD (AER) | USD 66–233 | USD 227–460 | Reusable |
| Boucheron 2022 (13) | France | Retrospective | manufacturer not specified | Ambu aS4C | Manual HLD | €196 | €192 | Single-use |

**Supplement Table 5. Economic comparison of reusable (RU) and single-use (SU) flexible cystoscopes**

*Abbreviations:* RU = reusable; SU = single-use; HLD = high-level disinfection; AER = automated endoscope reprocessor; LTS = low-temperature sterilisation.
